# *C. elegans*-inspired undulatory motion in a light-driven liquid crystal elastomer fiber

**DOI:** 10.1016/j.isci.2025.114617

**Published:** 2026-01-05

**Authors:** Yasaman Nemati, Ming Cheng, Zixuan Deng, Yanjun Liu, Arri Priimagi, Hao Zeng

**Affiliations:** 1 Faculty of Engineering and Natural Sciences, Tampere University, P.O. Box 541, 33101 Tampere, Finland; 2 Department of Electronic and Electrical Engineering, Southern University of Science and Technology, Shenzhen 518055, China; 3 State Key Laboratory of Advanced Displays and Optoelectronics Technologies, Center for Display Research, Department of Electronic and Computer Engineering, The Hong Kong University of Science and Technology, Hong Kong 999077, China

**Keywords:** Applied sciences, Biomaterials

## Abstract

Undulatory movement is widely observed in the animal kingdom, from snakes and earthworms to microorganisms. Mimicking such deformation is important in soft robotics in terms of locomotion control and navigation efficiency. However, realizing such motion at miniature scales in fluid environments remains difficult for soft actuators. Here, we present light-controlled undulatory motion inspired by *C. elegans*, realized in a millimeter-scale liquid crystal elastomer (LCE) fiber actuator under water. We use the sequential excitation of four laser beams to generate bimorphic actuation between two segments of the LCE, with a 45-degree phase delay between two consequent deformation phases. The actuator demonstrates stable figure-eight-like trajectories and directional steering through laser power modulation. Furthermore, the actuation performance scales with fiber length, providing amplitude tuning and demonstrating programmable control of locomotion.

## Highlights

• A centimeter-long liquid crystal elastomer fiber exhibits worm-like underwater locomotion
• Sequential four-beam excitations enable undulatory motion mimicking *C. elegans*
• The phase and segmental location of deformation can be programmed by light

## Graphical abstract

**Figure:**
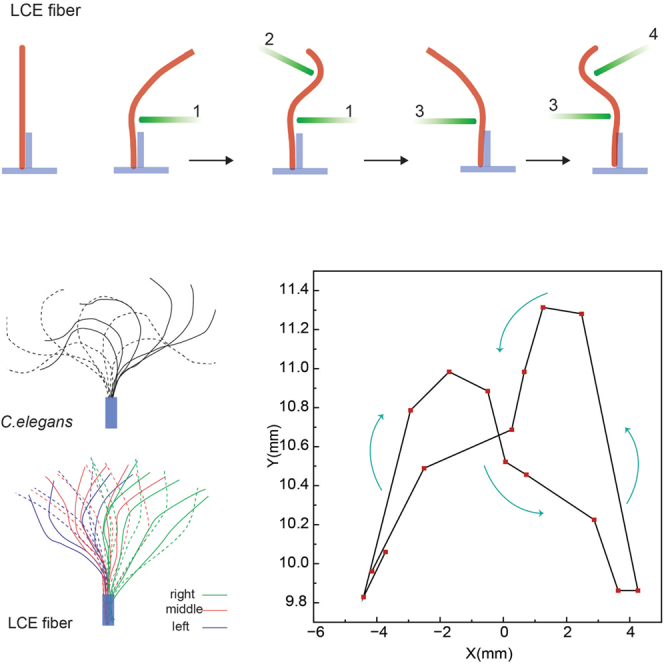

## Introduction

Nature provides elegant solutions for adaptive and efficient locomotion, inspiring the design of synthetic soft actuators that mimic biological movement strategies.^1^ Many limbless organisms, such as snakes and earthworms, achieve mobility through body deformations rather than relying on rigid appendages.^2^ Snakes, for example, generate propulsion through coordinated lateral undulations, exploiting surface friction for efficient movement.^3^ Earthworms, on the other hand, utilize peristaltic contraction and expansion of their body segments to navigate confined spaces.^4^ These biological systems benefit from multiple degrees of freedom, granted by their flexible musculature, which allows them to dynamically adapt their body shape and navigate complex surroundings. Additionally, their movement is facilitated by bimorph actuation, where the alternating activation of opposing muscle segments induces bidirectional bending.^5^^,^^6^^,^^7^ The synchronization between multiple biomorph actuators produces undulatory movement—a nonreciprocal movement strategy that enhances propulsion, optimizes hydrodynamic interactions, and improves energy efficiency.^8^^,^^9^

In contrast, human-made robotic systems typically rely on rigid-body actuators such as motors, cables, and piezoelectric elements, which require complex control mechanisms and external power sources.^10^^,^^11^ These constraints limit their adaptability, particularly at small scales. Light-responsive polymers offer a promising alternative due to their wireless control capabilities and ability to achieve multiple degrees of freedom in soft robotic actuation.^12^^,^^13^^,^^14^ Among them, liquid crystal elastomers (LCEs) have gained significant attention due to their ability to convert external stimuli, such as light^15^^,^^16^^,^^17^ or heat,^18^^,^^19^^,^^20^ into controllable mechanical deformations. Their tunable actuation properties make them particularly suitable for mimicking biological locomotion at small scales.^21^^,^^22^^,^^23^ Recent advances have positioned LCE fiber actuators as a versatile extension of this platform, offering high aspect ratios, large surface areas, and rapid, reversible deformation under various stimuli.^24^ Compared to LCE films and bulk structures, fibers provide greater structural flexibility and design freedom, enabling applications in artificial muscles, soft robotics, and adaptive textiles.^25^^,^^26^ Their actuation is typically driven by thermal heating, electrical Joule heating through embedded wires or liquid metals, or remote light-induced photothermal conversion using functional dopants. However, most existing fiber systems rely on global heating or tethered conductors, which limit spatial programmability and localized motion control, especially in fluidic environments.^19^^,^^27^^,^^28^

Realizing undulatory movement in synthetic systems necessitates coordinated motion between different body segments, and each of them must perform biomorphic actuation. This principle is evident in the undulatory movement of *Caenorhabditis elegans* (*C. elegans*), an organism that achieves forward propulsion through sequential dorsoventral muscle contractions.^29^ Different segments of *C. elegans’* body are actuated in sequence to generate traveling waves of deformation, facilitating effective navigation through surroundings.^30^ Despite its potential, this strategy has been rarely demonstrated in light-driven systems due to challenges in obtaining biomorph actuation and in the control of multiple degrees of freedom in deformation.^13^^,^^31^^,^^32^

In this work, we introduce a sequential laser-driven actuation approach for LCE fibers, enabling controlled, undulatory motion that mimics the natural locomotion of *C. elegans*. By modulating laser power, we demonstrate the ability to steer motion directionally, offering a simplified yet effective control strategy. This is achieved through localized photothermal bending, where the direction and amplitude of motion can be tuned by adjusting the intensity and sequence of laser inputs. Additionally, we show that fiber geometry, such as length, plays a role in determining displacement, offering further tunability in motion control.

## Results

### Bioinspired design and conceptual implementation of sequential actuation

**Figure 1:**
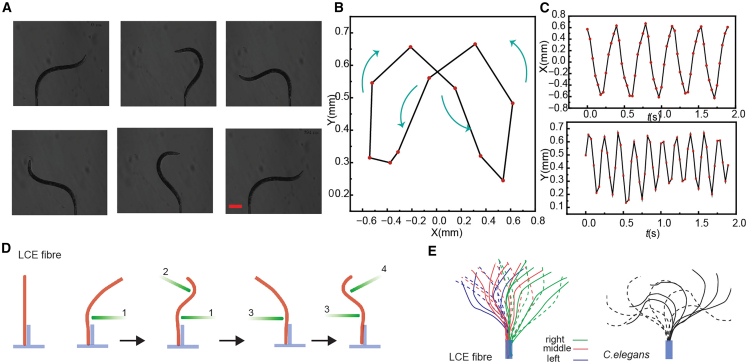
Bio-inspired robotic motion (A) Time-lapse snapshots of *C. elegans* motion over one cycle (adapted from [33]). Scale bars: 150 μm. (B) Corresponding tip trajectory in the X-Y plane. (C) X- and Y-displacements over multiple cycles. (D) Schematic of sequential laser actuation, where four laser beams modulate LCE fiber bending. (E) Schematic comparison of body deformation between *C. elegans* (right) and LCE fiber (left). (A) Reproduced from ref. 33 with permission from American Physical Society, copyright 2014.

Undulatory locomotion relies on sequential, wave-like deformations that generate net displacement through asymmetric body movements. Figure 1A presents a time-sequenced snapshot of *C. elegans* during one full locomotion cycle, illustrating its characteristic wave-like body deformation. These experimental images were obtained from Schulman et al., where *C. elegans* was partially immobilized at its tail using a micropipette setup, allowing precise tracking of its bending dynamics under controlled conditions.^33^ To quantify this motion, Figure 1B shows the corresponding trajectory of the worm’s tip in the X-Y plane, revealing the distinct looping motion. Figure 1C further analyses the tip displacement along the X and Y directions over multiple cycles, highlighting the time-dependent oscillatory motion of *C. elegans*.

To translate this biological motion strategy into a synthetic system, we developed a light-driven sequential actuation mechanism in an LCE fiber. LCEs can exhibit effective photothermal deformation in an aqueous environment, making them suitable for soft robotic applications that mimic biological locomotion. Leveraging this property, we designed an underwater actuation platform that sequentially bends the LCE fiber through localized laser excitation. Figure 1D schematically illustrates the stepwise activation process: four spatially distinct laser beams are directed onto the LCE fiber, each passing through an optical chopper with two blades to achieve sequential illumination. This dynamic light exposure induces localized heating, leading to reversible bending of the LCE fiber toward the illuminated region. As the optical chopper rotates, the lasers activate different parts of the LCE in succession and induce localized sequential bending, mimicking the undulatory pattern observed in *C. elegans*. Figure 1E schematically compares the body deformation over one cycle for both *C. elegans* (right) and the LCE fiber (left). The time-lapse of *C. elegans’* centerline illustrates the wave-like deformation of its body during locomotion. The deformation trajectories of the LCE fiber are mapped under sequential laser activation, revealing a pattern resembling that of *C. elegans*. By adjusting the power distribution between the laser beams, the fiber can be selectively actuated to move toward left, middle, or right, and the corresponding deformation profiles are extracted.

### Fabrication and photothermal characterization of light-responsive liquid crystal elastomer fibers

**Figure 2:**
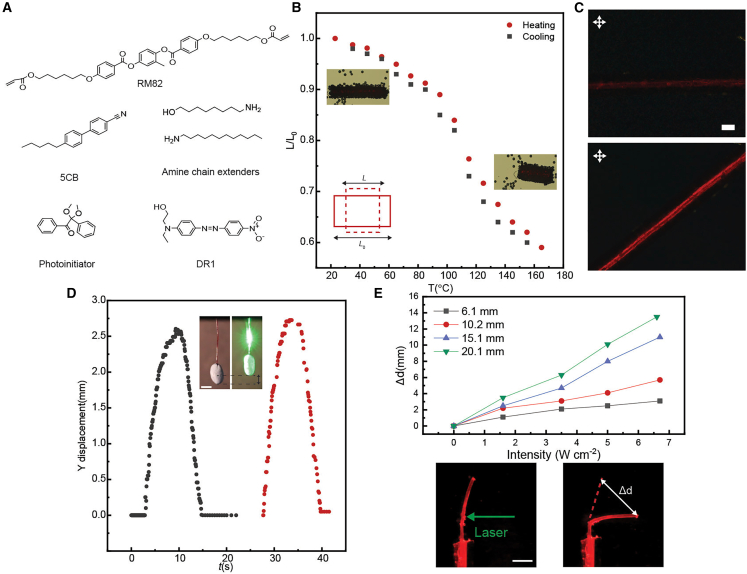
Mechanical properties (A) Chemical structures of the molecules utilized in the fabrication of the LCE fiber. (B) Heat-induced deformation during one thermal cycle. Inset: schematic drawing of the deformation of a planar-aligned LCE. L0: original length along the director. L: sample length after deformation. (C) Polarized optical micrographs of the LCE fiber at 0 and 45° angles between the molecular director and the polarizer. Scale bars: 0.2 mm. (D) LCE contraction (Y displacement) under light illumination. Irradiation conditions: 532 nm, 1.8 W cm^−2^. (E) Δd (Tip displacement) as a function of light intensity for 4 different LCE fiber lengths. Bottom: Photographs of the LCE fiber under light illumination. Scale bars: 3 mm.

Having established the biological inspiration and sequential actuation strategy, we next describe the fabrication and characterization of the LCE fibers. The fibers were fabricated by injecting a monomer mixture (Figure 2A) into 0.8-mm-diameter elastic silicone tubes. The mixture underwent oligomerization via aza-Michael addition reaction between liquid crystal diacrylates (RM82) and primary amines.^34^ To reduce the viscosity of the polymerizable precursor, non-polymerizable liquid crystal (5CB) was incorporated into the mixture.^35^ Photopolymerization was carried out under uniaxial stretching (100%) to achieve monodomain planar alignment with director coinciding with the long axis of the fiber. The details of the sample preparation are described in the STAR Methods section.

The thermomechanical response of the planar-aligned LCE fiber is shown in Figure 2B. The fiber exhibits contraction along the alignment direction upon heating. A 10% contraction is observed at 85 °C, increasing to approximately 40% at 165 °C. Figure 2C includes polarized optical microscopy images at 0° and 45° relative to the polarizer, confirming the uniform planar alignment of the LCE fiber. To endow the LCE with light sensitivity, Disperse Red 1 (DR1) was introduced into the LCE by immersing the fibers in a DR1–isopropanol solution. Dye diffusion from solution into the LCE imparted a red coloration of the LCE, making it responsive to blue-green light through photothermal heating. Figure 2D demonstrates the actuation kinetics upon light excitation (532 nm), showing the displacement of the fiber tip over time upon laser illumination. The uniaxially aligned LCE fiber undergoes controlled bending due to a thermal gradient across its thickness, causing deformation toward the incident light direction. This bending occurs because localized laser heating creates a thermal gradient across the fiber thickness. The resulting temperature difference produces differential thermal strain due to the anisotropic molecular alignment of the LCE, generating directional deformation.^36^ To quantify the extent of deformation, we measure the displacement of the fiber tip under laser excitation. The tip displacement (Δd) increases with rising input power and varies with fiber length. As shown in Figure 2E, longer fibers (20.1 and 15.1 mm) exhibit larger tip displacements compared to shorter ones (6.1 mm and 10.2 mm). Starting from the experiment shown in Figure 2E, all measurements were performed in an aqueous environment. To examine the effect of bath temperature on photothermal bending, we measured the bending angle of a single LCE fiber under laser excitation at three different water bath temperatures (20 °C, 35 °C, and 50 °C). As shown in Figure S1, the bending angle slightly increases with bath temperature. This is because a warmer bath temperature preconditions the LCE closer to its nematic-to-isotropic transition temperature range, reducing the additional energy required for deformation. Despite this, the overall actuation is still primarily driven by the localized photothermal gradient. These results confirm that while ambient temperature can tune the actuation, the principle of sequential undulatory motion remains robust.

### Sequential bimorph actuation enables directional control in liquid crystal elastomer fibers

**Figure 3:**
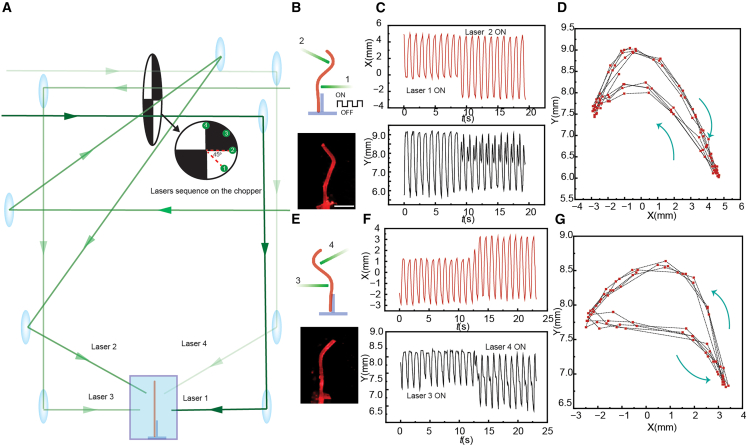
Directional control in sequential bimorphic actuation (A) Schematic diagram of the optical setup used. Inset: laser beam position at the chopper plane. (B) Top: Schematic illustration of sequential activation with Laser 1 and 2. Bottom: Experimental snapshot of the activated LCE fiber. (C) X- and Y-displacements of the fiber tip upon activation with Laser 1, followed by Laser 2. (D) Corresponding tip trajectory in the X-Y plane for activation with Laser 1 and 2. (E) Top: Schematic illustration of sequential activation with Laser 3 and 4. Bottom: Experimental snapshot of the activated LCE fiber. (F) X- and Y-displacements of the fiber tip upon activation with Laser 3, followed by Laser 4. (G) Corresponding tip trajectory in the X-Y plane for activation with Laser 3 and 4. Irradiation conditions: 532 nm, Laser 1 and 3: 4.7 W cm^−2^; Laser 2 and 4: 3.9 W cm^−2^. Scale bars: 3 mm.

To obtain undulatory motion, we employed a sequential laser-driven actuation strategy to achieve controlled bending of the LCE fiber. This motion is enabled by the photothermal gradient-driven response of the LCE, where bending occurs toward either side depending on the irradiation direction. The optical alignment and beam sequence are illustrated in Figure 3A. A single rotating optical chopper blade (Figure S2) was used to sequentially modulate the four spatially separated laser beams, introducing a phase delay that creates traveling bending waves along the fiber.

To systematically study this motion, the actuation sequence was divided into two laser pairs: Laser 1 and 2, and Laser 3 and 4 (Figures 3B and 3E). The phase delay between the sequential laser beams remains constant, i.e., 45°. The first pair initiates the primary bending phase, while the second reverses the deformation, completing one cycle of undulatory motion. Figure 3C presents the X- and Y-displacements of the fiber tip upon first activating with Laser 1, followed by activation with Laser 2, demonstrating the shift in displacement. The corresponding tip trajectory in the X-Y plane is shown in Figure 3D. Similarly, Figures 3F and 3G depict the motion when Laser 3 and Laser 4 are sequentially activated, further illustrating the bidirectional control of the fiber movement. This approach leverages the bimorph nature of LCE actuation, where the fiber exhibits bending in both directions due to the localized photothermal response. The introduction of a controlled time delay between laser activations allows the deformation to propagate as a traveling wave rather than a symmetric oscillation. This sequential actuation is essential for replicating the directional locomotion observed in organisms such as *C. elegans.* The periodic displacement observed in Figure 3C arises from the sequential activation of the laser beams. To exclude the possibility of water flow influencing the motion, we performed a control experiment with continuous single laser illumination. As shown in Figure S3, the fiber tip remained stable throughout the illumination period and only relaxed back when the laser was turned off. To assess the actuator’s response frequency, we tested the fiber under single laser excitation at different optical chopper frequencies (Figure S4). The results show that the LCE fiber maintains reliable actuation at higher frequencies; however, the displacement amplitude decreases as frequency increases, due to insufficient time for the actuator to complete its full deformation cycle. To evaluate the effect of fluid viscosity, we measured the tip displacement of the LCE fiber in water (1 mPa s), glycerol (1412 mPa s), and PEG400 (126 mPa s) under chopped laser actuation at frequencies of 1 Hz and 3 Hz (Figure S5). The results show that with increasing viscosity, the oscillation amplitude decreases, and this effect is amplified at higher actuation frequencies. Nevertheless, stable and repeatable motion was observed in all fluids, demonstrating that the sequential light-driven actuation strategy is robust across environments of varying viscosity. To further evaluate the dynamic response and stability, we characterized the long-term actuation stability of the fiber. As shown in Figure S6 the deformation remains stable over ∼5 min of continuous operation in water.

### Tunable undulatory motion and trajectory control through laser power and fiber length

**Figure 4:**
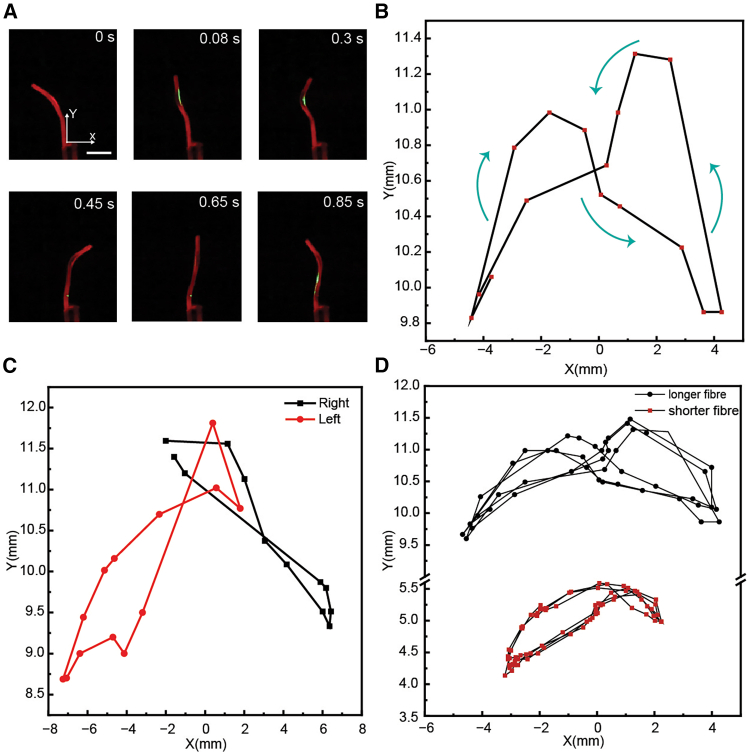
Trajectory control in undulatory motion (A) Time-lapse snapshots of the LCE fiber movement over one oscillation cycle. Irradiation conditions: 532 nm, Laser 1 and 3: 4.5 W cm^−2^; Laser 2 and 4: 3.7 W cm^−2^. Scale bars: 3 mm. (B) Corresponding tip trajectory in the X-Y plane. (C) Controlled directionality of motion by tuning the power of Laser 1 and 3. Right: Laser 1: 5.8 W cm^−2^; Laser 3: 4.5 W cm^−2^. Left: Laser 1: 4.5 W cm^−2^; Laser 3: 5.8 W cm^−2^. (D) Influence of fiber length on the tip displacement. Long fiber: 10 mm; Short fiber: 5 mm. Irradiation conditions: 532 nm, Laser 1 and 3: 4.5 W cm^−2^; Laser 2 and 4: 3.7 W cm^−2^.

Figure 4 presents the full actuation response of the LCE fiber under sequential activation of all four laser beams. Unlike in Figure 3, where the actuation was analyzed in separated laser pairs, here all four lasers are active in a complete cycle, enabling the fiber to undergo a continuous undulatory motion. Figure 4A provides time-lapse snapshots capturing the fiber deformation over one cycle (Video S1). The corresponding trajectory of the fiber tip in the X-Y plane is shown in Figure 4B, where the periodic motion closely resembles the natural wave-like movement of *C. elegans* (Figure 1B). To explicitly show the actuation response time, we present the X- and Y-positions of the fiber tip plotted versus time (Figure S7). The measured trajectory confirms a full cycle duration of approximately 1 s.

Video S1. Time-lapse video showing the full actuation cycle of the LCE fiber under the sequential activation of all four laser beams, related to Figure 4 The fiber exhibits a continuous undulatory motion, mimicking the wave-like locomotion of *C. elegans*.

Beyond demonstrating a complete undulatory cycle, our system also offers tunability through tuning the intensity of the laser beams. Figure 4C shows how adjusting the relative intensity of Laser 1 and 3 shifts the motion bias toward left or right. When the power of Laser 1 is increased, the fiber exhibits greater bending toward the right, effectively steering the trajectory (Video S2). Conversely, reversing the power steers the fiber to the left. This highlights the directional control capability of our actuation strategy, where asymmetry in laser power introduces an additional degree of freedom in motion control. The influence of fiber length on the undulatory motion is examined in Figure 4D. Two fibers, one short (5 mm) and one long (10 mm), are subjected to the same sequential irradiation. The longer fiber exhibits greater overall displacement, whereas the shorter fiber maintains more compact trajectories. This suggests that fiber length can be strategically tuned to optimize actuation performance depending on application needs, whether for extended-range locomotion or confined-space manipulation.

Video S2. Directional control of the LCE fiber by modulating the relative power of Lasers, related to Figure 4

## Discussion

Our study establishes a strategy for achieving *C. elegans* undulatory locomotion in a millimeter-scale LCE fiber through sequential, phase controlled light actuation. By employing four spatially separated laser beams with defined phase delays, we demonstrated figure-eight trajectories, directional steering through laser intensity modulation, and amplitude tuning via fiber length variation. These results highlight how spatiotemporally programmed photothermal actuation can reproduce key features of *C. elegans* locomotion in an aqueous environment. Building on these findings, future investigations could use theoretical modeling or finite element simulations to provide a more detailed understanding of the dimension-dependent behavior, capturing the interplay between photothermal actuation, curvature distribution, and elastic resistance. Such approaches would help optimize the actuator performance. To further enhance its bioinspired performance, future designs may incorporate structural features observed in natural systems. For instance, using tapered LCE fibers, where variations in thickness induce spatial differences in bending stiffness,^37^ could improve directional control and propulsion. Additionally, embedding modulus gradients along the fiber may enable spatially programmable flexibility, allowing for localized curvature modulation—resembling the function of biological musculature. These modifications could enhance wave propagation and energy transfer during actuation, thereby improving locomotion efficiency.

Another promising avenue is to extend the current design, in which one end of the fiber is fixed for stability, toward systems where both ends are free. Realizing such motion would require real time feedback to track fiber deformation and dynamically adjust the laser input, potentially enabling more biomimetic and autonomous locomotion. Incorporating a closed loop feedback system to adaptively control laser intensity and phase sequence could further enhance precision and allow programmable trajectories. Moreover, while the current study focuses on kinematic demonstration, the quantitative evaluation of the actuator’s mechanical output is another critical step. Previous studies on *C. elegans* employed micropipette-based setups to immobilize part of the body and deduce propulsive forces based on displacement against a calibrated sensor.^33^^,^^38^^,^^39^ A similar methodology could be adapted for LCE-based systems to directly measure the force and assess actuation efficiency. Likewise, systematic assessment of energy efficiency will be critical for advancing this concept toward practical application.

This work introduces a light-driven actuation strategy for LCE fibers, enabling controlled motion inspired by the undulatory locomotion of *C. elegans*. By harnessing photothermal gradient-driven bending and bimorph actuation, we achieve tunable and directional movement. The ability to steer deformation by adjusting laser power highlights the adaptability of this approach, making it a promising platform for bioinspired soft robotics in an aqueous environment.

### Limitations of the study

It is equally important to acknowledge the current limitations of our approach. The system depends on an external four-beam laser array and manually adjusted phase delays generated by a rotating optical chopper, both of which add complexity to operation. The lack of autonomous feedback control, along with the need for precise optical alignment, also hinders scalability and integration with additional actuators. Overcoming these challenges through autonomous beam-steering and monitoring systems will be crucial for enabling more versatile soft-robotic functionalities.

## Resource availability

### Lead contact

Further information and requests for resources should be directed to and will be fulfilled by the lead contact, Hao Zeng (hao.zeng@tuni.fi).

### Materials availability

This study did not generate new unique reagents.

### Data and code availability

• The original data and materials for this study have been deposited in Zenodo and are publicly available at https://doi.org/10.5281/zenodo.17737083. Accession numbers are listed in the key resources table.
• This article does not report any original code.
• Any additional information required to reanalyze the data reported in this article is available from the lead contact upon request.

## Acknowledgments

This work is financially supported by the European Union’s Horizon 2020 Research and Innovation Programme under the Marie Sklodowska-Curie Grant Agreement 956150 (STORM-BOTS) and by the 10.13039/501100000781European Research Council (Starting Grant project ONLINE, No. 101076207, for H.Z. and Consolidator Grant project MULTIMODAL, No. 101045223, for A.P.). We gratefully acknowledge the support from the 10.13039/501100002341Academy of Finland, the Funding for mobility cooperation with India, China or Germany (No. 349260), the Center of Excellence Life-Inspired Hybrid Materials (LIBER, No. 346107), and the Flagship Programme on Photonics Research and Innovation, No. 320165. We thank Matilda Backholm (Aalto) for the inspiring discussion.

## Author contributions

Conceptualization, H.Z.; methodology, Y.N., Z.D., and M.C.; original draft, Y.N.; writing – review and editing, H.Z., A.P., and Y.L.; resources, H.Z. and A.P.; supervision, H.Z. and A. P.

## Declaration of interests

There are no conflicts to declare.

## Declaration of generative AI and AI-assisted technologies in the writing process

During the preparation of this work, the author(s) used ChatGPT (OpenAI) for grammar editing and language refinement. After using this tool, the author(s) reviewed and edited the content as needed and took full responsibility for the content of the publication.

## STAR★Methods

### Key resources table

**Table:** 

REAGENT or RESOURCE	SOURCE	IDENTIFIER
**Chemicals, peptides, and recombinant proteins**		

1,4-Bis-[4-(6-acryloyloxyhexyloxy) benzoyloxy]-2-methylbenzene (99%, RM82)	SYNTHON	CAS: 125248-71-7
8-Amino-1-octanol	TCI	CAS: 19008-71-0
4-cyano-4′-n-pentylbiphenyl	TCI	CAS: 40817-08-1
dodecyl amine	TCI	CAS: 124-22-1
2,2-Dimethoxy-2-phenylacetophenone	Sigma-Aldrich	CAS: 24650-42-8

**Software and algorithms**		

Origin	OriginLab	OriginLab, Northampton, MA, USA
Tracker	Douglas Brown (Open Source Physics)	Brown, 2023

**Deposited data**		

The original data	Zenodo	https://doi.org/10.5281/zenodo.17737083

### Method details

#### LCE Fabrication

The LC mixture was formulated by dissolving 0.22 mmol RM82, 0.1 mmol 8-amino-1-octanol, 0.1 mmol dodecyl amine, 50 wt% 5CB, and 2.5 wt% 2,2-dimethoxy-2-phenylacetophenone at 80°C. This mixture was then injected into an elastic silicone tube while maintaining the temperature at 80°C for 10 minutes before gradually cooling to 45°C at a rate of 1°C per minute. To facilitate the aza-Michael addition reaction for oligomerization, the sample was kept at 45°C in an oven for 24 hours. Afterward, mechanical stretching was applied to the material before subjecting it to UV polymerization (365 nm, 180 mW cm^-2^) for 30 minutes. Once polymerization was complete, the silicone tube was dissolved in isopropanol, and the extracted LCE fiber was subjected to a curing process on a hot plate at 120°C, ensuring the evaporation of 5CB. Finally, the fiber was immersed in a DR1/isopropanol solution for dyeing. Characterization: Photographs and movies were captured with Canon 5D Mark III camera with 100 mm lens. Stress–strain curves were obtained by using a homemade tensile tester at a stretching speed of 0.05 mm/s. The alignment of the monodomain samples was characterized with a polarized optical microscope (Zeiss Axio Scope.A1) by imaging the samples with the director set to 0 and 45° angles between two crossed polarizers. A continuous laser (532 nm, 2 W, ROITHNER) was used for light excitation.

#### Data analysis

The quantitative data was extracted from the recorded movie with video analysis software (Tracker).

### Quantification and statistical analysis

Origin 2024 and Tracker were used for the measurements and statistical analysis. Details of statistical analyses are listed in figure legends.
