# Supplementary material for: C. elegans-inspired undulatory motion in a light-driven liquid crystal elastomer fiber
Source: iScience. 2026 Jan 5;29(2):114617. doi: 10.1016/j.isci.2025.114617 (PMC12856333; doi:10.1016/j.isci.2025.114617)
Supplement: Document S1. Figures S1–S7 [file mmc1.pdf]

iScience, Volume *29*

Supplemental information

C. elegans-inspired undulatory motion in a light-driven liquid crystal elastomer fiber

Yasaman Nemati, Ming Cheng, Zixuan Deng, Yanjun Liu, Arri Priimagi, and Hao Zeng

Supporting Information

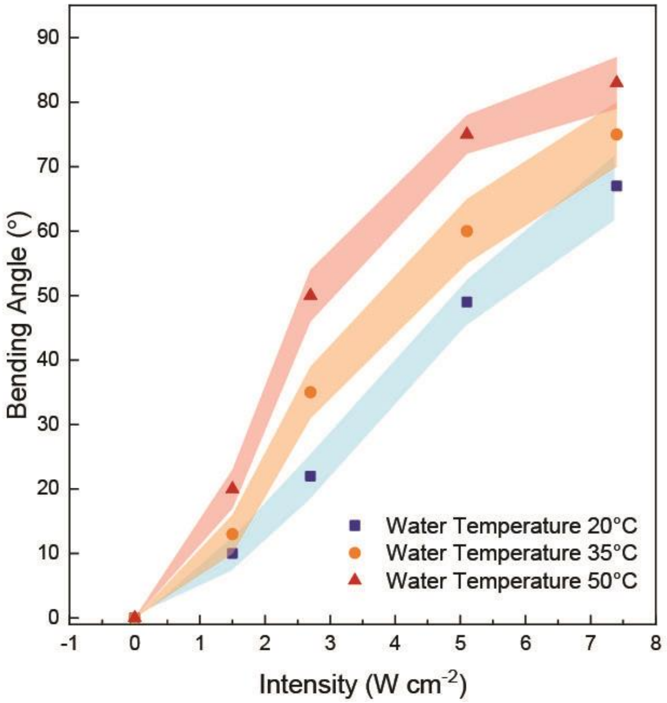

Fig. S1. Bending angle α as a function of light intensity for different water bath temperatures, Related to Figure 2.

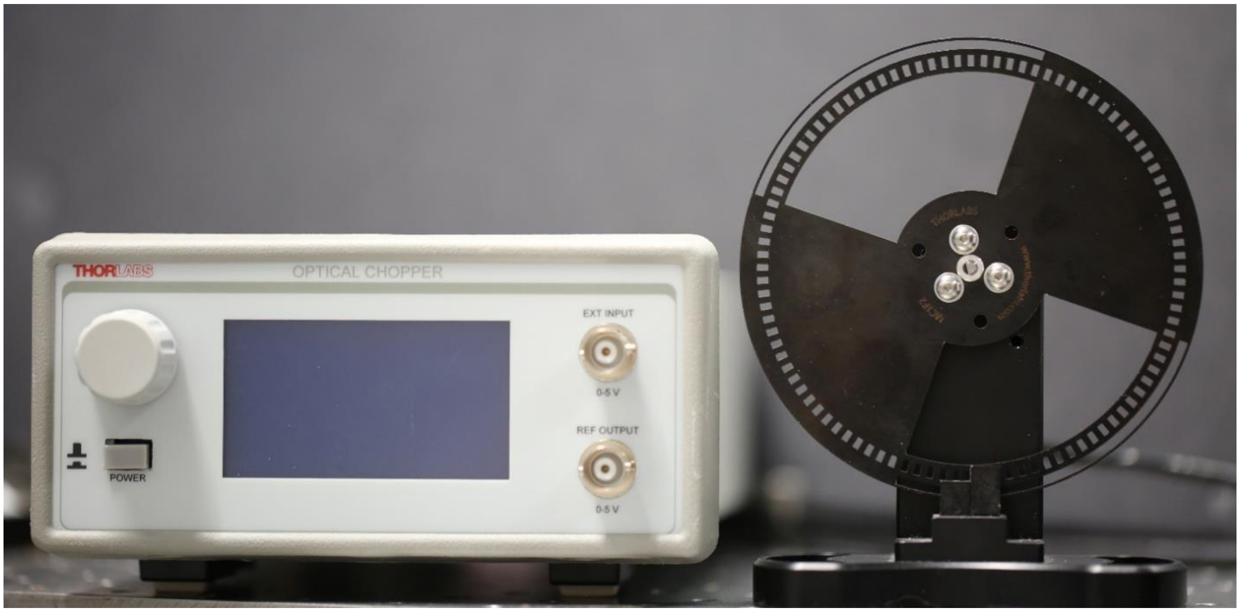

Fig. S2. Photograph of the optical chopper and controller used in the experimental setup, Related to Figure 3.

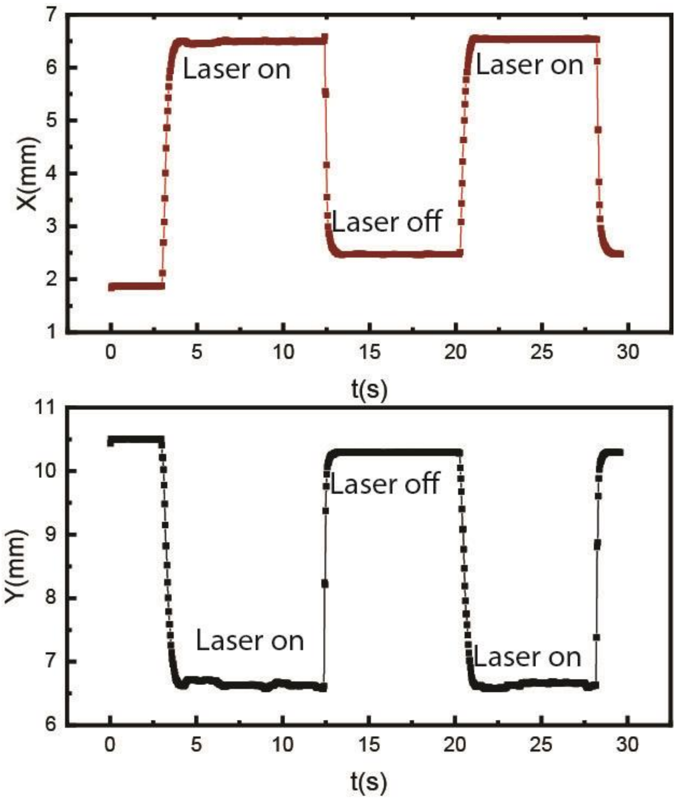

Fig. S3. Tip displacement of the LCE fiber under continuous single laser illumination, Related to Figure 2

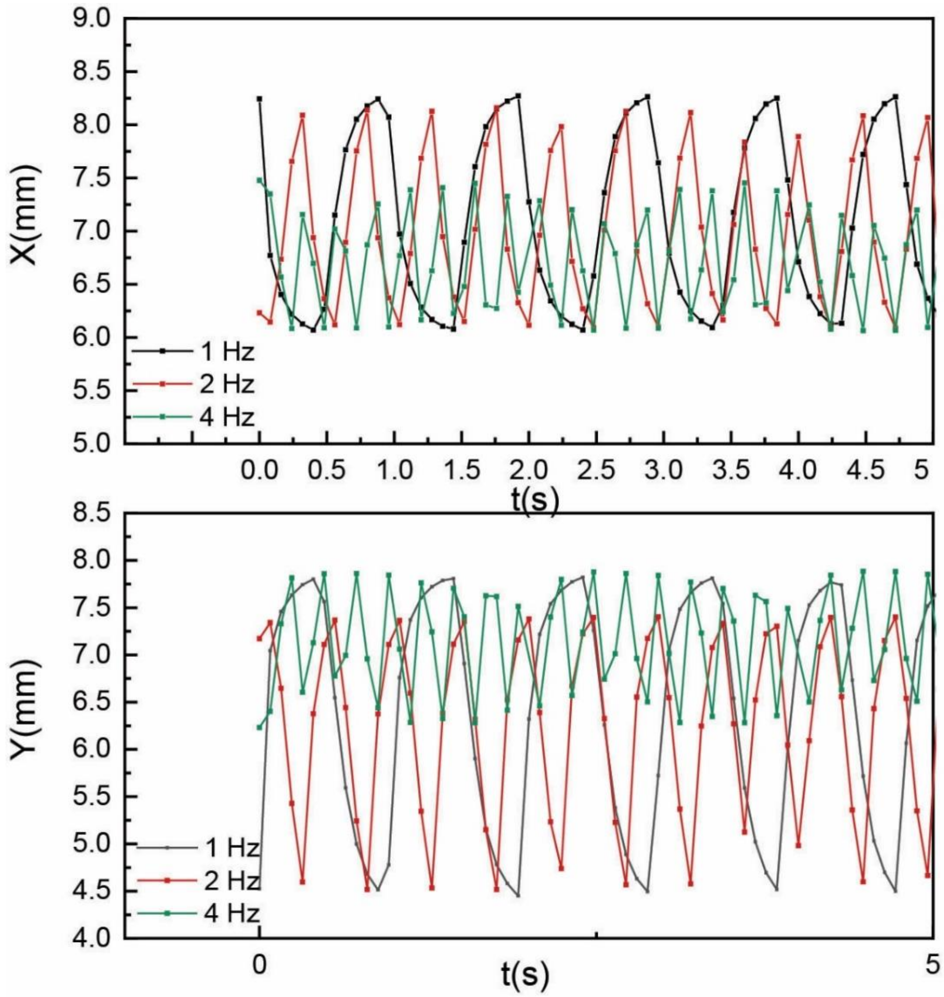

Fig. S4. X- and Y-displacements of the LCE fiber tip under single beam laser excitation at different modulation frequencies, controlled via an optical chopper, Related to Figure 2.

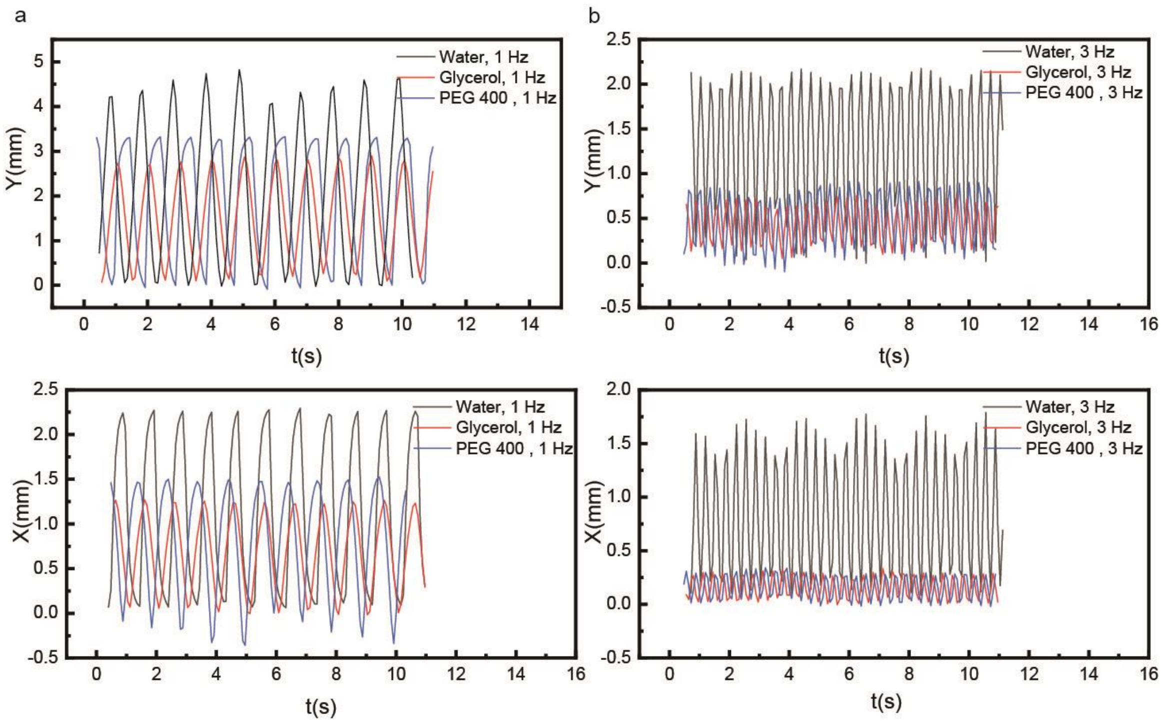

Fig. S5. Tip displacement of an LCE fiber actuated by a chopped laser beam in fluids of different viscosities at a) 1 Hz and b) 3 Hz chopping frequencies, Related to Figure 3.

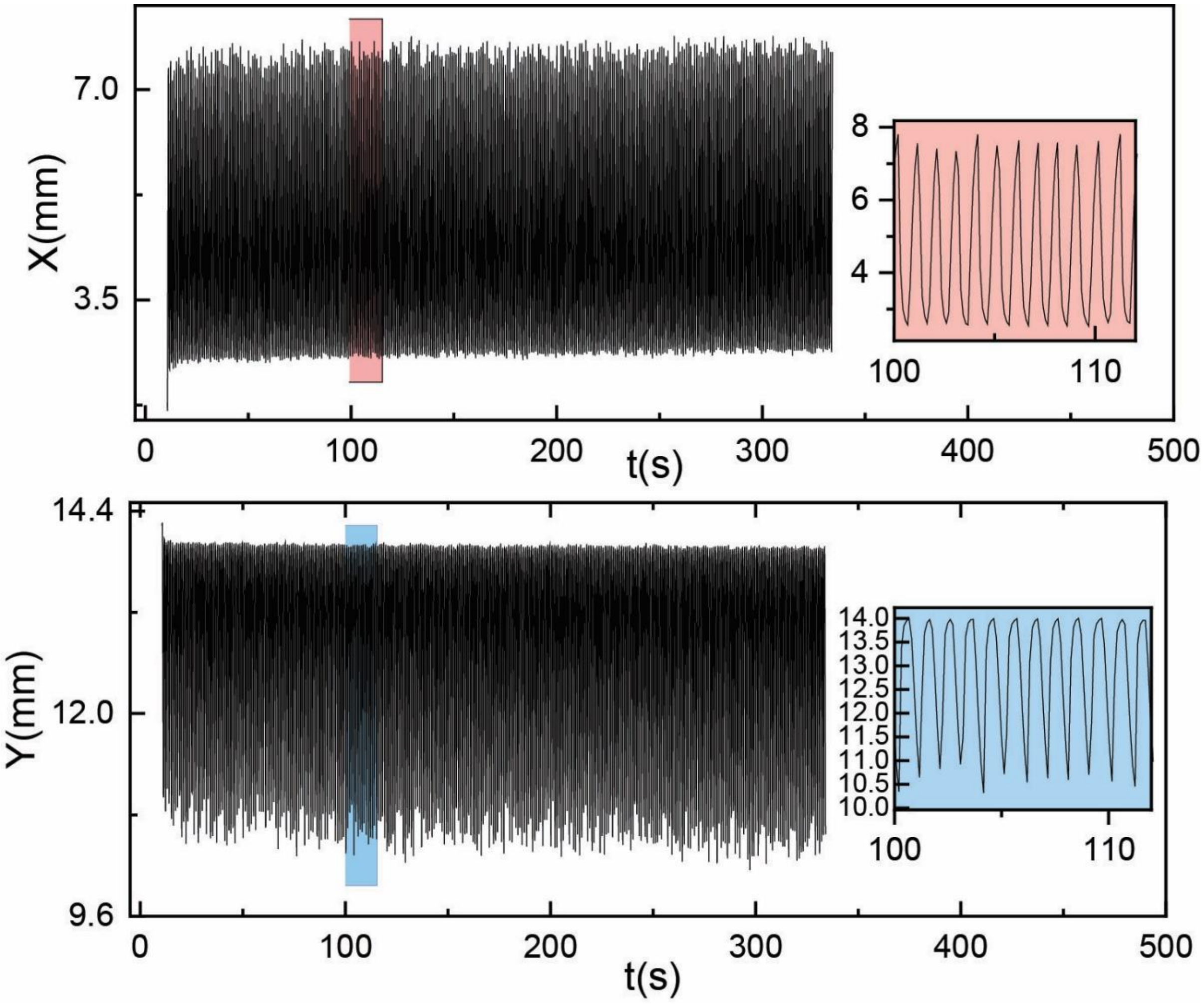

Fig. S6. X- and Y-displacements of the LCE fiber tip under single-beam laser excitation over ca. 5 minutes in an aqueous environment, Related to Figure 3.

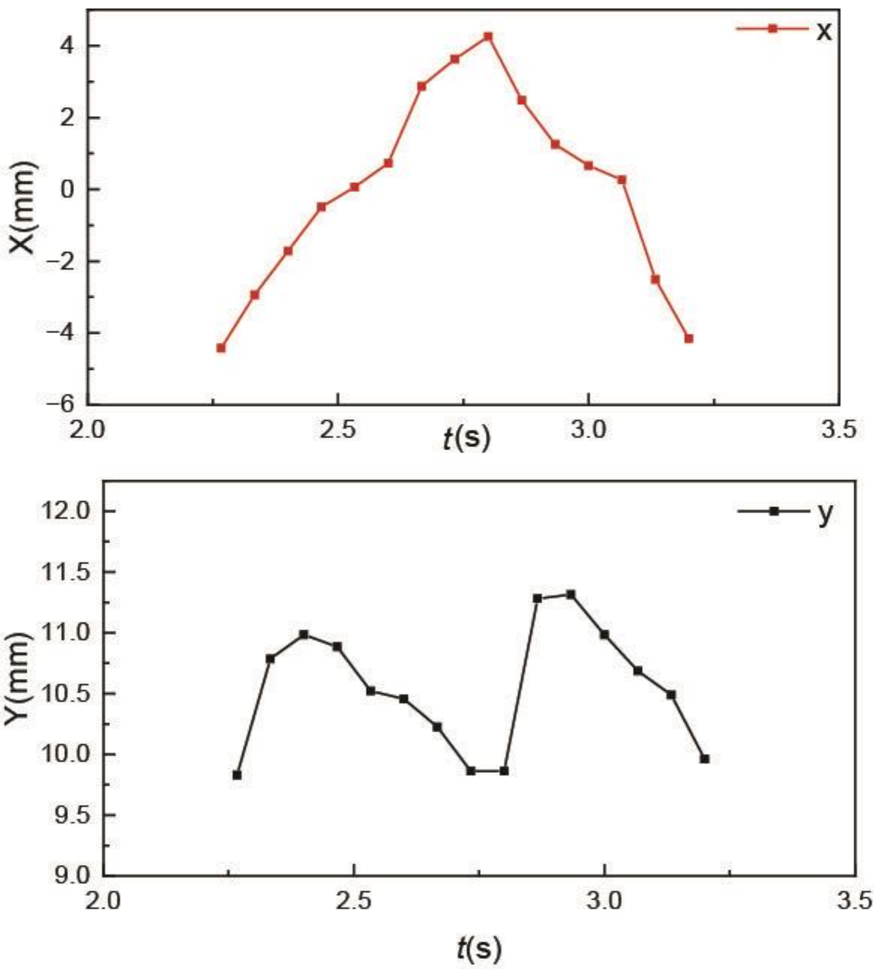

Fig. S7. X- and Y-displacement of the LCE fiber for one cycle of undulatory motion, Related to Figure 4.
